# Dyslexia and voxel-based morphometry: correlations between five behavioural measures of dyslexia and gray and white matter volumes

**DOI:** 10.1007/s11881-015-0102-2

**Published:** 2015-04-24

**Authors:** Peter Tamboer, H. Steven Scholte, Harrie C. M. Vorst

**Affiliations:** 1 Department of Psychology, Faculty of Social and Behavioural Sciences, University of Amsterdam, Amsterdam, The Netherlands; 2 Weesperplein 4, Room 218, 1018XA Amsterdam, The Netherlands; 3 Present Address: Overtoom 247B, 1054HW Amsterdam, The Netherlands

**Keywords:** Dyslexia, MRI, VBM, White matter, Gray matter, Cerebellum, Caudate nucleus

## Abstract

In voxel-based morphometry studies of dyslexia, the relation between causal theories of dyslexia and gray matter (GM) and white matter (WM) volume alterations is still under debate. Some alterations are consistently reported, but others failed to reach significance. We investigated GM alterations in a large sample of Dutch students (37 dyslexics and 57 non-dyslexics) with two analyses: group differences in local GM and total GM and WM volume and correlations between GM and WM volumes and five behavioural measures. We found no significant group differences after corrections for multiple comparisons although total WM volume was lower in the group of dyslexics when age was partialled out. We presented an overview of uncorrected clusters of voxels (*p* < 0.05, cluster size *k* > 200) with reduced or increased GM volume. We found four significant correlations between factors of dyslexia representing various behavioural measures and the clusters found in the first analysis. In the whole sample, a factor related to performances in spelling correlated negatively with GM volume in the left posterior cerebellum. Within the group of dyslexics, a factor related to performances in Dutch–English rhyme words correlated positively with GM volume in the left and right caudate nucleus and negatively with increased total WM volume. Most of our findings were in accordance with previous reports. A relatively new finding was the involvement of the caudate nucleus. We confirmed the multiple cognitive nature of dyslexia and suggested that experience greatly influences anatomical alterations depending on various subtypes of dyslexia, especially in a student sample.

## Introduction

Dyslexia has been described as a neurological disorder with a genetic origin characterised by poor reading and spelling abilities despite adequate intelligence, motivation and schooling. Dyslexia is persistent into adulthood, often regardless of remedial teaching during school days or other childhood interventions. Estimates of prevalence vary widely between 3 and 18 %. After decades of investigating the cognitive impairments of dyslexic people (e.g. Ramus & Ahissar, 2012), an important question in recent years has been whether structural and functional abnormalities in the brain can be identified in relation to dyslexia.

In this study, we address the issue of structural alterations in the brain in terms of anatomical brain morphology. A much-applied technique for analysing anatomical structures in the brain is voxel-based morphometry (VBM) (Ashburner & Friston, 2000; Wright et al., 1995), which specifies gray matter (GM) and white matter (WM) densities of separate voxels. Unfortunately, in VBM studies of dyslexia, many findings failed to be replicated or were rendered insignificant (statistically) by corrections for multiple comparisons. As a result, there is much discussion about the generalisability of findings.

Besides this discussion, some findings appear to be consistent across studies and much has already been learned. Two meta-analytical studies were reported in 2012, examining local GM alterations in relatively small samples of dyslexic adults. A coordinate-based meta-analysis (Richlan, Kronbichler, & Wimmer, 2012) of nine VBM studies reporting 43 foci of GM reduction and 2 foci of GM increase in dyslexic readers (total sample sizes, 134 dyslexic and 132 non-impaired mostly adult readers, 11–41 years) resulted in the convergence of GM reductions in only two relatively small areas: one in the right superior temporal gyrus and one in the left superior temporal sulcus. No significant differences in whole brain GM or WM volume were reported. An activation likelihood estimation meta-analysis (Linkersdörfer et al., 2012) of nine VBM studies reporting 62 foci of GM reduction in dyslexic readers (total sample sizes, 139 dyslexic and 138 non-impaired mostly adult readers) resulted in the convergence of six clusters in bilateral temporo-parietal and left occipito-temporal cortical regions and in the cerebellum bilaterally. Again, no significant differences in whole brain GM or WM volume were reported.

Seven studies were incorporated in both meta-analyses (Brambati et al., 2004; Brown et al., 2001; Eckert et al., 2005; Hoeft et al. 2007; Kronbichler et al., 2008; Steinbrink et al., 2008; Vinkenbosch, Robichon, & Eliez, 2005). In the analysis by Richlan et al., a study by Raschle, Chang, & Gaab (2011) was excluded because the participants were prereading kindergarteners with a family history of dyslexia but without diagnosis of dyslexia, and a study by Pernet et al. (2009a) was excluded because they failed to find direct group differences. In the analysis by Linkersdörfer et al., a study by Silani et al. (2005) and a study by Menghini et al. (2008) were not included. The reported coordinates of the areas of convergence were not exactly the same which may be the result of slightly different inclusion criteria of the studies. The largest cluster in the study by Linkersdörfer et al. was found in the left fusiform gyrus extending into the left inferior temporal gyrus, while Richlan et al. found a cluster in the left superior temporal sulcus. Both studies reported a cluster in the right superior temporal gyrus, but Linkersdörfer et al. reported four additional areas in the left and right supramarginal gyrus and in the left and right cerebellum, which failed to reach meta-analytical significance in the study of Richlan et al.

Besides these meta-analyses, findings from functional imaging studies are also relevant for interpreting brain anatomy of dyslexics. Two meta-analytic studies of functional overactivation and underactivations in dyslexics were performed in recent years (Richlan, Kronbichler, & Wimmer, 2009, 2011). In Linkersdörfer et al., the results of these studies were used to analyse overlap between structural and functional deviations with additional activation likelihood estimation meta-analyses of imaging studies. Conjunction analyses of the meta-analyses revealed an overlap in the left cerebellum and left fusiform gyrus. Summarising all meta-analytic results, it became clear that some areas are involved in dyslexia with a high degree of certainty. However, the number and size of the areas which survived meta-analytical significance thresholds are small compared to the number and size of all areas reported in the smaller samples of the separate studies. That many areas did not survive significance thresholds does not automatically imply that these are irrelevant for dyslexia.

Support for the significance of some areas that did not survive significance thresholds can be found in the study of Pernet et al. (2009a). In this study, no significant group differences were found in a large sample of 38 dyslexics and 39 non-dyslexics. However, most of the uncorrected *p* values pointed to areas in accord with previous findings. Moreover, this study reported various significant correlations between GM volumes (41 loci) and behavioural measures (phoneme deletion, irregular word spelling, pseudoword reading), across groups and/or between groups. The 41 loci were found in three main territories: the cerebellum; the ventral visual cortex; and several parts of (mainly) left and dorsal hemispheric brain areas such as superior frontal, medial parietal and superior temporal areas. Thus, in a relatively large sample using correlational analyses, many more areas could be significantly related to dyslexia than in the meta-analyses. In a second study by Pernet et al. (2009b), two predictors of dyslexia were found using a classification approach: the right lentiform nucleus and the right cerebellar declive with dyslexics falling either above or below the control group’s 95 % confidence interval boundaries.

In summary, much has been learned about brain anatomy in dyslexia, but two main questions remain under debate: why are more significant alterations identified in studies with smaller samples than in studies with larger samples or in meta-analytical studies, and why are correlational analyses more efficient in identifying anatomical alterations than group analyses? First of all, no explanations can be derived from gender differences. Both in the study by Pernet et al. and in most meta-analytic studies, a large majority of the participants were male. Thus, possible GM differences between dyslexic males and dyslexic females as were observed by Evans et al. (2013) could not explain the differences. On the other hand, the inclusion of a relatively small portion of female dyslexics in the samples might have had an effect on the power of group differences between dyslexics and controls.

A plausible explanation for the fact that most of the direct group differences in separate studies failed to be significant seems to be that the samples in these studies were relatively small (on average, 15 dyslexics and 15 non-dyslexics), resulting in a lack of power. However, it is then strange that other findings in separate studies were significant, while in meta-analytical studies or in a large sample (Pernet et al.), most findings failed to reach significance. Pernet et al. pointed out that, given the common use of small samples in MRI studies, “individual variability may have been under- or overestimated… biasing results towards significance in some studies and not in others” (p. 2288). Relatively high individual variability in groups of dyslexics is well established, and the common explanation is that dyslexia is a multiple cognitive deficit. VBM results can then easily be affected by individual variation in perception and cognition in dyslexia (Eckert et al., 2005). Analyses of individual profiles has also resulted in the suggestion that subtypes of dyslexia should be distinguished (e.g. Ramus et al., 2003). Although there is an ongoing discussion about subtypes, there is strong support and consistency for at least two subtypes of dyslexia (Bosse et al. 2007; Di Filippo & Zoccolotti, 2012; Heim et al., 2008): a phonological subtype and a visuo-attentional subtype. Furthermore, different samples of different ages may induce different results because it was found that many GM density differences between dyslexics and controls, in general, result from differences in reading experience (Krafnick et al., 2014; Clark et al., 2014).

In a recent VBM study by Jednoróg et al. (2014) with 46 dyslexic and 35 control children (mean age 10 years), the dyslexic children were split into three subtypes based on various cognitive deficits (phonological, rapid naming, magnocellular/dorsal, auditory attention shifting). Comparing VBM contrasts for the whole group revealed significantly reduced local GM volume for dyslexics compared to controls in the left inferior frontal gyrus—an area that failed to reach significance in the meta-analyses. Most importantly, comparing VBM contrasts of separate subtypes with all other groups revealed different loci of reduced and/or increased GM volume in various areas. This underlines that in VBM studies with small samples, some subtypes of dyslexia may have been overrepresented or underrepresented. It explains why more significant alterations are identified in studies with small samples than in studies with large samples or in meta-analytical studies and why correlational analyses between behavioural measures and local GM volumes are more efficient in identifying anatomical alterations than analyses of group differences. However, it is highly important to realise that the anatomical differences between subtypes of dyslexia in the study by Jednoróg et al. were found in young children while the participants in the meta-analyses were mainly adults. In the meta-analysis of functional MRI studies (Richlan, Kronbichler, & Wimmer, 2011), various differences exist between dyslexic children and adult dyslexics. It is unknown how these differences influence anatomical differences with age. In general, it is also unknown whether age differences have influenced results in the adult samples used for the meta-analyses which varied between 11 and 41 years old.

The aim of the present study was to enlarge the probability of finding significant anatomical differences between dyslexics and non-dyslexics. We used a large sample of 37 dyslexic and 57 non-dyslexic students. This sample was used in a previous study (Tamboer, Vorst & Oort, 2014a) in which dyslexics and non-dyslexics were identified on the basis of various cognitive measures. In a second previous study (Tamboer, Vorst & Oort, 2014b), the same sample was used for the identification of five cognitive factors related to dyslexia (phonology, spelling, short-term memory, rhyme words/confusion and whole-word reading/complexity). Thus, we took into account that dyslexia is characterised by various cognitive deficits as much as possible. Compared to the studies by Pernet et al. (2009a) who used three behavioural measures and Heim et al. (2008) who used three cognitive factors, we were able to specify relations between cognition and anatomical alterations further. First, we applied analyses of group differences using whole-brain VBM instead of analysing only a priori-determined areas in a region-of-interest (ROI) analysis, because in previous studies, brain abnormalities were reported in various brain areas, and we did not want to run the risk of missing relevant regions by limiting our analyses to ROIs. Second, we applied correlational analyses between loci of GM volume alterations and five cognitive factors. Third, to account for effects of gender, age and handedness, we exploratively performed various additional analyses with these variables as covariates.

## Methods

### Subjects and procedure

In this study, 37 dyslexic students (six men; six left-handed; mean age 20.61 years, SD 1.53 years) and 57 non-dyslexic students (seven men; eight left-handed; mean age 20.33 years, SD 1.14 years) participated. All participants were first-year psychology students at the University of Amsterdam, and most of them were female as most psychology students in the Netherlands are female. All students were native Dutch speakers, were raised in the Netherlands and had 12–13 years of school education at a school in the Netherlands. All students were free from medical or psychiatric diseases and had no history of sensory deficits or head trauma. ADHD was assessed with a short self-report questionnaire, which included 46 questions about attention, concentration and hyperactivity. A mean score and standard deviation on this questionnaire were calculated in a larger group of more than 1000 students. The groups of dyslexics and non-dyslexics did not differ on ADHD symptoms, and no student of the present sample had a score higher than one standard deviation above the average of the total sample of more than 1000 students. Handedness was assessed with a short self-report questionnaire, which included questions about writing hand, general hand preference and 20 specific questions. There were no students with inconsistent reports which could indicate being ambidextrous. All students who participated in this study gave informed written consent and were debriefed afterwards. All participants had the option to choose between acquiring participation points required for the first year of study or a financial reward. This study was approved by the ethics committee at the University of Amsterdam.

### Neuropsychological assessment of dyslexia

This sample was acquired from a sample of students who participated in a previous study (Tamboer et al., 2014a). In that study, dyslexia and non-dyslexia were assessed using three sources of information: a history of language difficulties, a self-report of language difficulties and a test battery.

A history of language difficulties consisted information about persistent language difficulties at school, dyslexic family members and various test results which were assessed during school days. Some students had a formal diagnosis of dyslexia. In the Netherlands, a formal diagnosis of dyslexia can be acquired only from official institutes of dyslexia by specialists in diagnosing dyslexia and is considered to be very reliable.

A self-report of dyslexia was assessed with an extended questionnaire that consisted of two parts. The first part consisted of 30 general questions or statements, such as ‘Are you dyslexic?’, ‘Did you experience difficulties with learning to read and/or to spell during school days?’, ‘I do not like that English sounds differently than it is written’. The second part consisted of 140 specific statements which aim to acquire information about specific language difficulties. The questionnaire was designed according to a 7 × 5 × 4 facet design. There are seven subscales representing different aspects of language, each consisting of 20 statements with seven response categories. Here, we give examples for the seven subscales: *reading*: ‘Sometimes I skip a letter, which results in reading a different word’; *writing*: ‘Sometimes I forget to write down a syllable’; *speaking*: ‘While speaking, I sometimes exchange similar words’; *listening*: ‘I hear a story exactly like someone tells it’; *copying*: ‘When I copy out a text, I sometimes exchange letters with similar sounds’; *dictating*: ‘I make mistakes in dictation, because I do not hear the correct sounds’; and *reading aloud*: ‘When reading aloud, I sometimes repeat a part of the text’. All statements can also be categorised into five subscales representing *sounds*, *letters*, *words*, *sentences* and *text*. A leading thought during the creation of these statements was that four typical mistakes might distinguish dyslexics from others: *skipping* (*forgetting*), *adding*, *changing* and *exchanging*. These typical mistakes represent various kinds of visual, attentional, auditory or phonological confusion. Furthermore, we accounted for the possibility that these typical mistakes become more prominent when complexity increases, as is the case with pseudowords, for instance.

The test battery consisted of nine language-related tests and a short-term memory test. Together, these ten tests covered all of the known symptoms of dyslexia, such as phonological awareness, rapid naming, attentional/visual processing and short-term memory.

1. *Dutch dictation* (auditory) aims to measure spelling abilities in the Dutch language (ten sentences, maximum score 10 × 4 = 40). Each sentence consisted of at least two difficult words.
2. *English dictation* (auditory) aims to measure spelling abilities in the English language (ten sentences, maximum score 10 × 2 = 20). It can be assumed that Dutch students are familiar with ordinary English words that we used. Each sentence consisted of at least two difficult words.
3. *Missing letters* (auditory) also aim to measure spelling abilities in the Dutch language (ten sentences, maximum score 10 × 2 = 20), but in a slightly different way. For each sentence, two words are repeated while these words are shown on the computer screen with a few letters left out of the word.
4. *Pseudowords* (auditory) aim to measure spelling abilities of pseudowords—non-words that sound like real words (30 words, maximum score 30). Participants have to decide whether the non-words that they hear are spelled correctly on the computer screen. Usually, pseudowords are administered the other way around by participants reading the words aloud themselves. We changed this because it would be practically impossible to have all students in private sessions for this way of testing.
5. *Sound deletion* (auditory) aims to measure phonological abilities (20 words, maximum score 20). Participants have to decide whether the difficult Dutch words that they hear are pronounced correctly, and if not, which letter is missing or has been added (there is a choice between three words). For example, the word ‘fietsenstalling’, which means bicycle shed, is read out as ‘fiestenstalling’. The possible answers are as follows: ‘fietsentalling’, ‘fiestensalling’ and ‘fiestenstalling’.
6. *Spoonerisms* (auditory) also aim to measure phonological abilities (20 words, maximum score 20). A spoonerism is a word that consists of two existing smaller words and still consists of two small existing words when the first letters of both small words are exchanged. For example, participants hear the word ‘kolen-schop’ which has to be altered to ‘scholen-kop’.
7. *Incorrect spelling* (visual) is the third test in our study that aims to measure spelling abilities in the Dutch language, again in a different way (40 words, maximum score 40). All words are flashed on a computer screen for 50 ms. Participants have to decide whether the words are spelled correctly or not.
8. *Dutch*–*English rhyme words* (visual) aim to measure the ability to recognise similar-sounding nouns in Dutch and English (40 words, maximum score 40). Dutch–English word pairs are shown on a computer screen with the Dutch words on the right. Participants have to decide whether the words rhyme with each other or not. Typical confusion may arise in this test because the non-rhyming items have the same vowels, such as ‘Deep-Reep’.
9. *Letter order* (visual) aims to measure the ability to read words as a whole (20 sentences, maximum score 20 × 2 = 40; time limit of 5 min). We created 20 sentences based on the same principle: the order of the letters of the words was changed, apart from the first and last letters. The words in the sentences are more difficult towards the end of the test. The sentences have to be typed in with all words correctly spelled. There are no words that consist of typical dyslexic spelling difficulties.
10. The *short*-*term memory test* aims to measure the capacity of short-term memory. We used the concept of digit span: the number of digits that a person can retain and recall. There are four subtests: numbers and letters, both forward and backward. And, each subtest consists of 24 series: 6 of 4, 6 of 5, 6 of 6 and 6 of 7 items for the subtest numbers and letters forward and 6 of 3, 6 of 4, 6 of 5 and 6 of 6 items for the subtest numbers and letters backward. The numbers and letters are presented one by one, for 1 s each on a computer screen. The participants have to retype these numbers and letters after the last one of a series has been presented. About half of all series consist of some typical difficulties for dyslexics, either phonological, visual or both. For example, a typical phonological confusion is between the numbers 7 and 9 which resemble each other phonologically in Dutch (zeven/negen). Typical visual confusions are between the numbers 6 and 9 and the letters [m] and [w]. The letters [p], [d] and [b] resemble each other phonologically as well as visually.
  For the selection of non-dyslexics in the present study, we required that they had no history of language difficulties, that they had no self-report of dyslexia or related difficulties and that they had no more than three of 12 test scores below average and, if so, not lower than a half standard deviation below average.
  For the selection of dyslexics in the present study, we required that dyslexics were identified as dyslexic with all sources of information. Thus, the dyslexics in the present study had a history of language difficulties, provided a self-report of dyslexia and were identified as dyslexic with the battery of 12 tests. The identification process of the test battery was based on various discriminant and logistic regression analyses using both sum scores and single test items as predictors. The use of single test items as predictors was based on the idea that sometimes, only specific test items are representative of typical difficulties for dyslexics.
  Table 1 provides group differences between dyslexics and non-dyslexics on all tests and questionnaires related to dyslexia. Non-dyslexics performed significantly better than dyslexics on all tests and questionnaires of dyslexia (most *p* < 0.001), except on sound deletion, although there was a significant difference in the larger original sample.

**Table 1:** Behavioral measures (Z-scores) (tests and questionnaires of dyslexia, factors of dyslexia, intelligence and school grades)

	Dyslexics		Non-dyslexics		*T*	*p* value
	(*N* = 37)		(*N* = 57)			
	*M*	SD	*M*	SD		
Tests of dyslexia						
Dutch dictation	−1.04	(0.95)	0.38	(0.64)	8.64	<0.001
English dictation	−0.91	(1.42)	0.27	(0.72)	5.30	<0.001
Missing letters	−0.58	(0.95)	0.40	(0.66)	5.85	<0.001
Pseudowords	−0.55	(1.07)	0.44	(0.64)	5.55	<0.001
Sound deletion	−0.30	(1.24)	0.08	(0.74)	1.85	0.067
Spoonerisms	−0.73	(1.12)	0.36	(0.75)	5.37	<0.001
Incorrect spelling test	−1.06	(1.22)	0.33	(0.88)	6.38	<0.001
Dutch–English rhyme words	−0.91	(1.30)	0.23	(0.68)	5.57	<0.001
Letters exchanging	−0.63	(1.03)	0.33	(0.81)	5.02	<0.001
Digit span	−0.94	(1.15)	0.40	(0.88)	6.23	<0.001
Self-report questions						
Self-report reading	−1.16	(0.62)	0.48	(0.89)	9.76	<0.001
Self-report writing	−1.19	(0.71)	0.45	(0.79)	10.16	<0.001
Self-report speaking	−0.80	(0.84)	0.17	(0.98)	4.99	<0.001
Self-report listening	−0.46	(0.81)	0.18	(1.06)	3.08	0.003
Self-report copying	−0.96	(0.67)	0.36	(0.86)	7.89	<0.001
Self-report dictating	−0.98	(0.71)	0.43	(0.90)	8.05	<0.001
Self-report reading aloud	−0.95	(0.74)	0.35	(0.97)	6.94	<0.001
Factors of dyslexia						
Spelling	−1.10	(0.81)	0.37	(0.59)	10.23	<0.001
Phonology	−0.51	(1.46)	0.12	(0.78)	2.74	0.007
Short-term memory	−0.60	(1.07)	0.23	(1.02)	3.78	<0.000
Rhyme/confusion	−0.53	(1.37)	0.12	(0.71)	3.02	0.003
Whole-word processing/complexity	−0.55	(0.97)	0.42	(0.77)	5.38	<0.001
Intelligence						
Vocabulary	−0.52	(0.87)	0.14	(1.08)	3.07	0.003
Verbal analogies	−0.47	(0.90)	0.18	(1.04)	3.14	0.002
Numeric progressions	−0.38	(0.78)	0.03	(1.01)	2.10	0.038
Conclusions	−0.05	(0.90)	0.15	(1.03)	0.92	0.360
Speed of calculation	−0.53	(0.62)	0.27	(1.16)	4.34	<0.001
Hidden figures	−0.19	(0.90)	0.13	(1.06)	1.46	0.148
Raven progressive matrices	−0.26	(1.14)	0.06	(1.10)	1.31	0.195
Final course grades from school						
Dutch	6.45	(0.86)	7.11	(0.79)	3.56	0.001
English	6.47	(1.14)	7.09	(1.25)	2.35	0.021
Other languages	6.58	(0.62)	7.13	(0.70)	3.61	0.001
Mathematics	6.52	(1.39)	6.53	(0.80)	0.06	0.949
Other courses	6.97	(0.57)	7.06	(0.67)	0.65	0.515

### Neuropsychological assessment: assessment of five factors of dyslexia

In a second previous study (Tamboer et al., 2014b), we used factor analysis on the whole dataset to identify cognitive measures of dyslexia. With principal component analyses, five independent factors of dyslexia could be distinguished. These factors explained 60 % of the variance of the tests and questionnaires which were also used for the identification study. In a confirmatory factor analysis, we found a root-mean-square error of approximation (RMSEA, which is a measure of the error of approximation of the model-implied covariance matrix to the population covariance matrix and should be lower than 0.05) of 0.03 with *p* = 0.88 which means that the null hypothesis of a close fit cannot be rejected.

1. Factor *spelling* (15.7 % of variance)
  High factor loadings for this factor were found for the tests ‘Dutch dictation’, ‘English dictation’, ‘missing letters’ and ‘incorrect spelling’ and for questions related to spelling.
2. Factor *phonology* (10.8 % of variance)
  High factor loadings for this factor were found for the tests ‘pseudowords’ and ‘sound deletion’ and for questions related to phonology.
3. Factor *short*-*term memory* (12.1 % of variance)
  High factor loadings for this factor were found for the test ‘short-term memory’ and for questions related to memory.
4. Factor *rhyme*/*confusion* (11.3 % of variance)
  High factor loadings for this factor were found for the test ‘Dutch–English rhyme words’ and for questions related to phonological, visual, attentional and/or auditory confusion in language.
5. Factor *whole*-*word processing*/*complexity* (10.3 % of variance)
  High factor loadings for this factor were found for the test ‘letter order’ and for questions related to complexity.
  Table 1 provides group differences between dyslexics and non-dyslexics for the five factors of dyslexia. The mean scores are presented as Z-scores which were acquired in the previous study from a total sample of 495 students. *T* tests were performed for the groups of the present study. Non-dyslexics performed significantly better than dyslexics on all factors of dyslexia (all *p* < 0.01). The difference on the factor spelling was much larger than the differences on the other factors, which implies that spelling difficulties are the most prominent difficulties for this group of dyslexics. Furthermore, we should note that dyslexics showed higher variances for all factors, especially on the phonology and rhyme/confusion factors.

### Neuropsychological assessment: assessment of intelligence

We assumed that the intelligence of all participants was within the normal range, because all were students who had finished the highest level of school education. Nevertheless, we investigated differences in intelligence between the 37 dyslexics and 57 controls with various tests of intelligence and with final course grades from school. These grades can vary between 1 and 10. A score of six or higher means that a student has passed the course. Compared to the non-dyslexic group, the dyslexic group had lower final school grades for Dutch language (*p* = 0.001), English language (*p* = 0.021) and other languages (*p* = 0.001), but not for mathematics and other courses. Six subtests of a cognitive battery based on the structure-of-intellect model of Guilford and Raven’s Progressive Matrices were used for measures of intelligence. The mean performance of the whole group of about 1000 students on all tests was about one standard deviation above average compared to the normative standard in a total population. In this sample, dyslexics performed worse than non-dyslexics in four subtests of a cognitive battery based on the structure-of-intellect model of Guilford: vocabulary (*p* = 0.003), verbal analogies (*p* = 0.002), speed of calculation (*p* < 0.0005) and numeric progressions (*p* = 0.038), but no differences were found on the subtests conclusions, hidden figures and on Raven’s Progressive Matrices (see also Table 1).

### Voxel-based morphometry

We performed a voxel-based morphometry (VBM) analysis to find differences in GM volume in brain areas over subjects. For this, we acquired a structural scan for each of the subjects. From the first 45 subjects (15 dyslexics), we obtained one T1 recording per subject (3D T1, Turbo Field Echo, voxel size = 1 mm^3^, field of view (FOV) = 256^2, 160 slices, flip angle (FA) = 8, echo time (TE) = 3.78, repetition time (TR) = 8.24), using a 3.0-T Philips Achieva scanner. From the last 49 subjects (22 dyslexics), we obtained three T1 recordings per subject (3D T1, Turbo Field Echo sequences, voxel size = 1 mm^3^, FOV = 256^2, 160 slices, FA = 8, TE = 3.81, TR = 8.24), using a 3.0-T Philips Achieva scanner. We used the average image. After conducting *t* tests, we found no differences in head coils or noise between the two samples.

Data were analysed with FSL-VBM (Good et al., 2001), using FSL (Smith et al., 2004). First, structural images were brain-extracted. Next, tissue-type segmentation was carried out using FAST4 (Zhang, Brady, & Smith, 2001). The resulting GM and WM partial volume images were then aligned to MNI152 standard space using the affine registration. The resulting images were averaged to create a study-specific template, to which the native GM images were then non-linearly re-registered with a method that uses a B-spline representation of the registration warp field (Andersson et al. 2007a, b; Rueckert et al., 1999). The registered partial volume images were then modulated (to correct for local expansion or contraction) by dividing the Jacobian of the warp field. The modulated segmented images were then smoothed with an isotropic Gaussian kernel with a kernel of 4 mm. The segmented WM and GM volumes were used to determine the total amount of GM and WM per subject.

### Statistical analyses

Group differences in GM volume were calculated with permutation-based non-parametric testing (using a gap test) to find voxels that differed between subjects with and without dyslexia. The resulting clusters of differences in GM volume were corrected for multiple comparisons using random field theory with a cluster threshold of *t* >2.3 and a reliability of *p* < 0.05 for extend of the cluster. Alternatively, we thresholded the resulting contrast looking for clusters of 200 connected voxels with a *p* value lower than 0.05 in the VBM analysis (identical to Rouw & Scholte, 2010). The choice of 200 connected voxels is large, but choosing a smaller threshold would lead to tendencies with a decreasing reliability to be relevant. This second analysis was performed with two purposes. First, in the case of finding only a few or even no significant results, we wanted to determine clusters which could be considered tendencies and which could then be compared with previous findings. Second, we wanted to explore to what degree the GM volume in these clusters could be related to behavioural constructs.

The local demeaned GM volumes and the total GM and WM volumes were correlated (Pearson) in SPSS with five demeaned factor scores representing five cognitive aspects of dyslexia. These correlations were computed for all subjects and within groups, thus resulting in 15 comparisons for each cluster of GM. The correlations were corrected for multiple comparisons using the false discovery rate (FDR).

To account for the effects of age, gender and handedness, we recalculated group differences using an ANOVA analysis with these variables as fixed factors and recalculated the correlations partialling out these variables.

## Results

### Dyslexic versus non-dyslexic subjects

No differences were observed between dyslexics and non-dyslexics in total GM volume (0.66 vs. 0.67; *T*(92) = 1.24, *p* = 0.22) and total WM volume (0.57 vs. 0.59; *T*(92) = 1.18, *p* = 0.24). Voxel-by-voxel GM volume comparisons revealed no significant differences in local GM volumes between dyslexics and non-dyslexics after correcting for multiple comparisons. Uncorrected clusters (*p* < 0.05, cluster size *k* > 200) are presented in Table 2. Three clusters of increased GM volume for dyslexics were found in the left posterior cerebellum (and a small part of the occipital fusiform gyrus), the left inferior parietal lobe (parts of angular and posterior supramarginal gyrus) and in the right superior temporal gyrus. Eight clusters of reduced GM volume for dyslexics were found in the left and right caudate nucleus, the right inferior temporal gyrus, the right angular gyrus, the left parietal operculum (insula), the right frontal lobe and in the left and right middle frontal gyrus.

**Table 2:** Brain areas that represent tendencies (statistical trends) of GM alterations (uncorrected for multiple comparisons, not significant, *p* < 0.05, *k* > 200 voxels)

Region	MNI coordinates			Voxels
	(centre of gravity)			
	*X*	*Y*	*Z*	
Dyslexics > non-dyslexics				
L posterior cerebellum (occipital fusiform gyrus)	−32	−76	−23	749
L inferior parietal lobe (parts of angular and posterior supramarginal gyrus)	−57	−53	42	215
R superior temporal gyrus	50	−7	−12	306
Non-dyslexics > dyslexics				
L caudate nucleus	−14	15	7	402
R caudate nucleus	10	14	8	377
R inferior temporal gyrus	58	−53	−21	434
R angular gyrus	53	−52	22	1516
L parietal operculum (insula)	−47	−27	23	261
R frontal lobe	20	39	40	1705
R middle frontal gyrus	38	47	−12	320
L middle frontal gyrus	−41	49	−8	538

### Correlations between behavioural constructs and local gray matter volumes

**Fig. 1:**
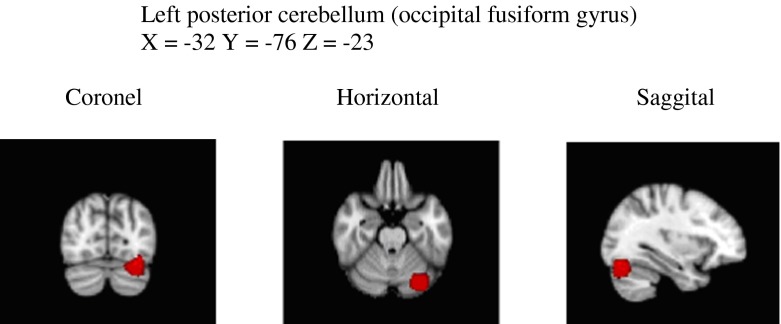
Left posterior cerebellum (occipital fusiform gyrus) (*X* = −32, *Y* = −76, *Z* = −23)

**Fig. 2:**
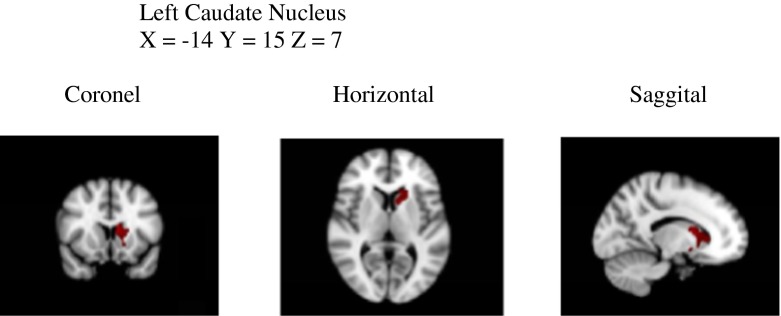
Left caudate nucleus (*X* = −14, *Y* = 15, *Z* = 7)

**Fig. 3:**
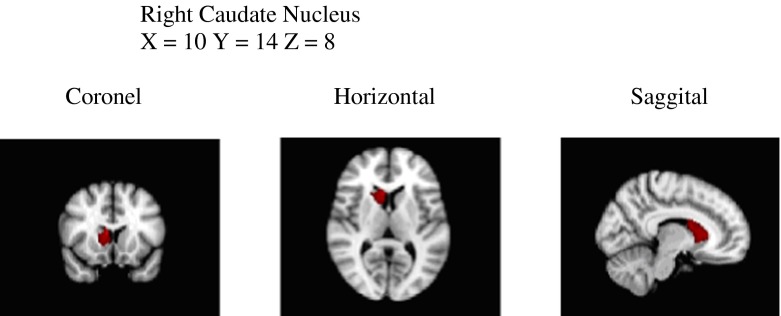
Right caudate nucleus (*X* = 10, *Y* = 14, *Z* = 8)

**Fig. 4:**
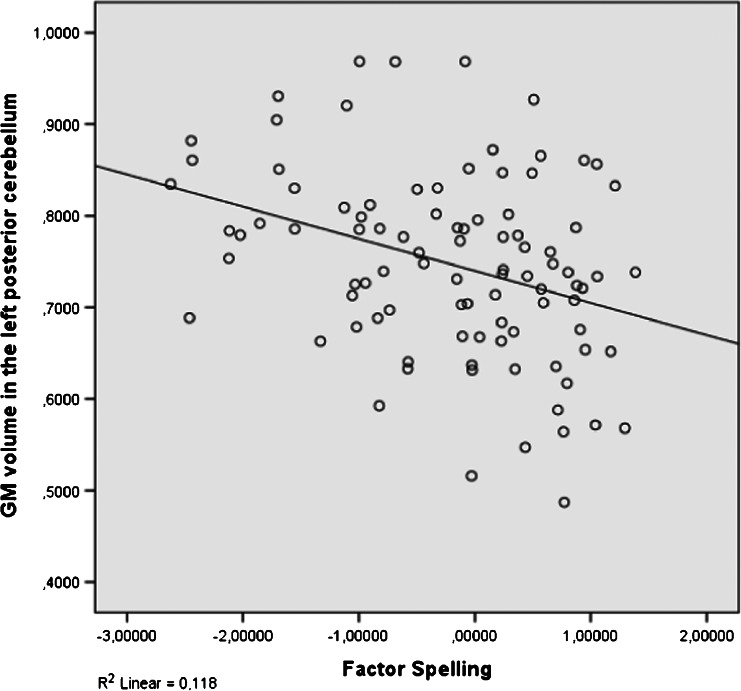
Correlation between spelling and GM volume in the left posterior cerebellum (all subjects: *N* = 94)

**Fig. 5:**
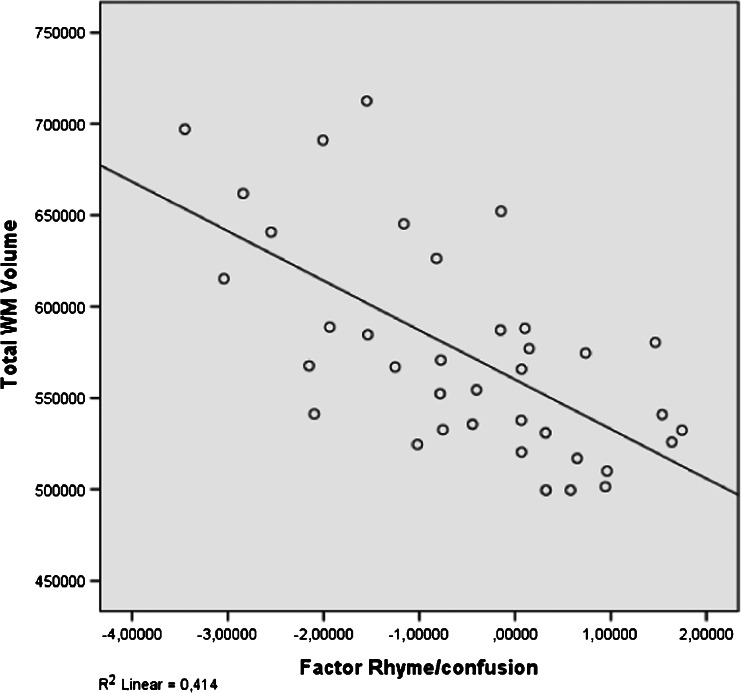
Correlation between rhyme/confusion and total WM volume (subjects with dyslexia: *N* = 37)

**Fig. 6:**
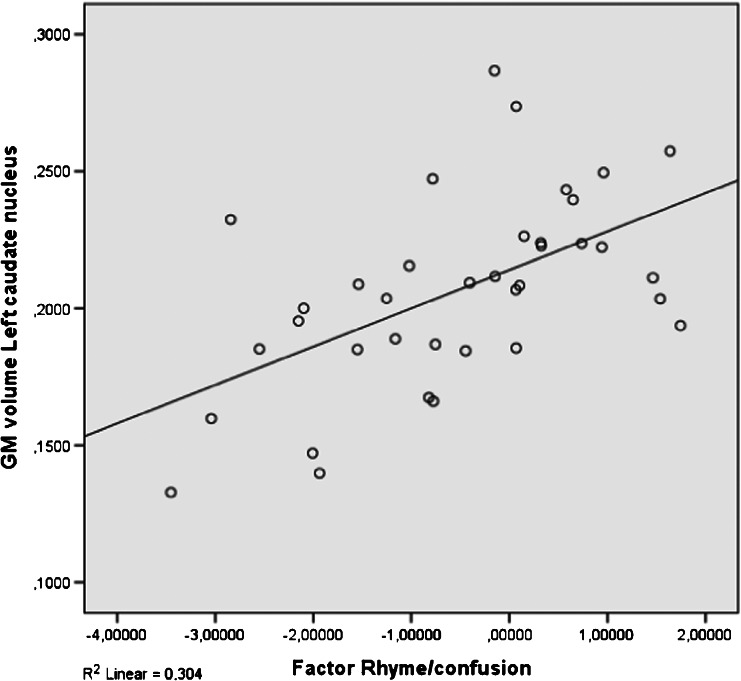
Correlation between rhyme/confusion and the left caudate nucleus (subjects with dyslexia: *N* = 37)

**Fig. 7:**
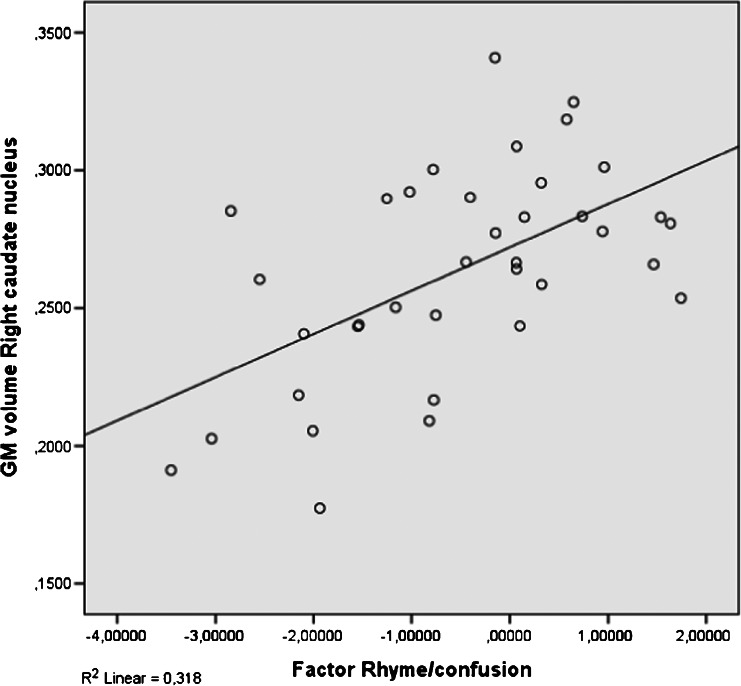
Correlation between rhyme/confusion and the right caudate nucleus (subjects with dyslexia: *N* = 37)

Five factors of dyslexia were correlated (Pearson) with total GM, total WM and 11 clusters of local GM volumes. All correlations were calculated for all subjects and within groups and corrected for multiple comparisons (5 × 13 × 3 = 195 comparisons) using FDR, which resulted in four significant correlations. The relevant brain areas are presented in Figs. 1, 2 and 3. Scatterplots are presented in Figs. 4, 5, 6 and 7. A negative correlation (*r* = −0.34, *p* = 0.034) for all subjects was observed between the factor spelling and GM volume in the left posterior cerebellum (and a small part of the occipital fusiform gyrus). This means that poor performances on spelling tasks correlated with increased GM volume in this area. Within the group of dyslexics, a negative correlation (*r* = −0.64, *p* = 0.003) was observed between the factor rhyme/confusion and total WM volume. This means that poor performances on tasks related to rhyme/confusion correlated with increased total WM volume. Two positive correlations within the group of dyslexics were observed between the factor rhyme/confusion and GM volume in the left caudate nucleus (*r* = 0.55, *p* = 0.026) and in the right caudate nucleus (*r* = 0.56, *p* = 0.027). This means that poor performances on tasks related to confusion correlated with reduced GM volume in the left and right caudate nucleus. A few other correlations within the group of dyslexics were found but did not reach significance after FDR correction, while no uncorrected correlations larger than 0.3 were found within the group of non-dyslexics.

### Age, gender and handedness

To account for the effects of age, gender and handedness, we performed various analyses. Chi-square tests revealed that the groups of dyslexics and non-dyslexics did not differ regarding proportion of men versus women and proportion of left- versus right-handedness. A *t* test revealed no group differences for age. Two-way ANOVAs for group differences on total GM and WM volume revealed two main effects for gender with men having larger GM and WM volumes (both *p* < 0.001) and a main effect of dyslexia when age was partialled out (with age explaining 2 % of variance of WM and older students having increased WM volume) with dyslexics having reduced WM volume compared to non-dyslexics (*p* = 0.015). However, we found no interaction effects between dyslexia and age, gender or handedness. Correlational analyses partialling out age, gender and handedness revealed the same significant correlations (FDR corrected) as in the analyses without covariates. A negative correlation (*r* = −0.34, *p* = 0.045) for all subjects was observed between the factor spelling and GM volume in the left posterior cerebellum. Within the group of dyslexics, a negative correlation (*r* = −0.55, *p* = 0.049) was observed between the factor rhyme/confusion and total WM volume. Two positive correlations within the group of dyslexics were observed between the factor rhyme/confusion and GM volume in the head of the left caudate nucleus (*r* = 0.59, *p* = 0.026) and in the head of the right caudate nucleus (*r* = 0.61, *p* = 0.033). No uncorrected correlations larger than 0.3 were found within the group of non-dyslexics.

## Discussion

This study confirmed two main conclusions from previous studies. First, a large sample did not result in significant group differences between dyslexics and non-dyslexics in local GM volumes. Second, correlations between cognitive measures and local GM volumes provide significant findings. This study supported the view of dyslexia as a multiple cognitive deficit and related various cognitive measures to local alterations in GM volume. Areas of GM alterations were considered tendencies if not significant. However, most findings were in accordance with previous reports supporting the view that all anatomical brain findings in dyslexia contribute to the discussion in an accumulating way.

In summary, we found no significant differences in total GM volume, total WM volume and local GM volumes between the groups of dyslexics and non-dyslexics after corrections for multiple comparisons. However, dyslexics showed significant lower total WM volume than non-dyslexics when effects of age were partialled out. We found three areas of increased GM volume for dyslexics and eight areas of reduced GM volume for dyslexics. These areas were thresholded by 200 connected voxels with a *p* value lower than 0.05 and were considered tendencies. Most of these areas were reported in previous studies. In contrast with the absence of significant differences of local GM volumes, we found significant correlations between behavioural measures and alterations in GM volume, also when effects of age, gender and handedness were partialled out. An important finding was that no correlations were observed within the group of non-dyslexics, while three correlations were observed within the group of dyslexics and one in the whole group. We should also note that the present sample mainly consisted of females while in most previous studies, samples mostly consisted of males. The largest areas found in this study were observed in the right hemisphere consistent with a recent finding (Evans & Flowers, 2013) that anatomical differences between female dyslexics and female non-dyslexics are more prominent in the right hemisphere compared to differences between male dyslexics and non-dyslexics.

### Rhyme/confusion and the caudate nucleus

Within the group of dyslexics, a factor rhyme/confusion representing performances on the test ‘Dutch–English rhyme words’ and questions related to phonological, visual, attentional and/or auditory confusion in language correlated positively with GM volume in both the left and right caudate nucleus. Reductions of GM volume in the caudate nucleus have been reported only once in one of the first VBM studies of dyslexia (Brown et al., 2001) also finding these reductions bilaterally. Remarkably, hyperactivations of dyslexics in the left caudate were reported in an fMRI study, however without anatomical alterations (Hoeft et al. 2007). Numerous studies showed that the caudate nucleus is implicated in several executive control functions (Brovelli et al., 2011; Grahn, Parkinson, & Owen, 2008; Lewis et al., 2004; Provost, Petrides, & Monchi, 2010; White, 2009), language functions such as language switching in bilinguals (Luk et al., 2012), second language learning (Hosoda et al., 2013; Tan et al., 2011) and suppression of irrelevant words (Ali et al., 2009). In relation to these findings, the relation between the caudate nucleus and the test Dutch–English rhyme words, which requires fast switching between languages, makes perfect sense. This is also the case with the confusion reported via self-report questions, which represented typical mistakes, such as exchanging letters within words and exchanging words within sentences—mistakes that might result from impaired cognitive control of attention. We think that the possibility exists that reduced GM volume in the caudate nucleus in relation to rhyme/confusion might represent a more fundamental dysfunction of dyslexic people, which might encompass various difficulties of confusion, such as exchanging letters or words. A recent study by Hachmann et al. (2013) seems to support this. These researchers found that impaired performances on tasks of short-term memory are explained by impaired serial order processing rather than impaired storage.

### Confusion, spelling and white matter density

Dyslexics as a group had reduced WM volume compared to non-dyslexics when age was partialled out. In contrast, within the group of dyslexics, rhyme/confusion correlated negatively with total WM volume, meaning that the dyslexics who are more severely impaired regarding rhyme/confusion have larger total WM volume. Apparently, different behavioural constructs have different effects on WM volume. It is generally assumed that WM volume represents connectivity in the brain. Regarding dyslexia, it has been hypothesised that dyslexics suffer from impaired connectivity (e.g. Steinbrink et al., 2008). Based on the results of the present study, we alternatively hypothesise that confusion may result from too much connectivity in some areas. Connection efficiency has also been investigated using diffusion tensor imaging (DTI), which quantifies the relative diffusivity of water in a voxel into directional components. However, while the relation between WM volume and the so called fractional anisotropy (FA) remains somewhat unclear, a meta-analysis of DTI studies (Vandermosten et al., 2012) only resulted in reduced FA values (mainly in a left temporoparietal region which hosts two WM tracts: the left arcuate fasciculus and the left corona radiate). But, higher FA values were reported in the splenium, the posterior end of the corpus callosum which connects the left and right cerebral hemispheres (Frye et al., 2008; Odegard et al., 2009). This might be viewed as support for the idea that confusion correlates with too much connectivity. But actually, the main thing which is supported by all these results regarding WM volume alterations is the complex nature of dyslexia. This is emphasised even more, for instance, by theories of increased WM gyral depth in the brains of dyslexics (Casanova et al., 2010). The idea is that reduced WM volume is the result of broader gyri or any other change in the thickness of the cortex, involution of sulci and/or complexity of cortical folding.

### Spelling and the cerebellum

Better performances on spelling tasks correlated with reduced GM volume in the left posterior cerebellum (and a small part of the left occipital fusiform gyrus) in the whole group of students. In the meta-analysis by Richlan et al. (2012), cerebellar abnormalities did not survive significant thresholds. In the meta-analysis by Linkersdörfer et al. (2012), reduced GM volumes were found bilaterally in the cerebellum, although located more anterior than the area of increased GM volume in the cerebellum in this study. Despite somewhat different coordinates, this seems to be in contrast with each other. However, in a study by Pernet et al. (2009b), using a classification approach, the right cerebellar declive was one of the two best predictors of dyslexia, with dyslexics falling either above or below the control group’s 95 % confidence interval boundaries. Remarkably, our cluster of increased GM volume in the left cerebellum was found more or less on the opposite site of the cluster found by Pernet et al. In the study by Jednoróg et al. (2014), increased GM volume for one subtype of dyslexics was reported in the left cerebellum/lingual gyrus, while in the same area, reduced GM volume was reported for another subtype of dyslexics. It becomes even more puzzling when we compare these findings with findings of increased symmetry in dyslexics as opposed to non-dyslexics showing more right GM than left GM (Rae et al., 2002) or with findings of differences in asymmetry between dyslexics with and without a phonological deficit (Leonard et al., 2001). One alternative explanation for inconsistent findings in the cerebellum might be that the cerebellum can be difficult to segment because of volume averaging in the folia in comparison to cerebral cortex.

Another explanation can be derived from the following. Generally, spelling is one of the most commonly reported symptoms of dyslexia. However, in schools, poor-performing children also receive extra training when they are not dyslexic. This might explain why spelling correlates with the cerebellum across groups. The cerebellum is associated with skill acquisition and automatisation and specifically with aspects of language processing (Hodge et al., 2010; Murdoch, 2010). In dyslexia, impaired functioning of the cerebellum is associated with impaired reading fluency and motor deficits (Nicholson & Fawcett, 2007). These findings seem to support increased GM volume in the cerebellum from training in spelling abilities, instead of reductions in GM volume. A strong argument in favour of these learning effects related to dyslexia is that cerebellar findings seem to depend on the age of the subjects. For example, a VBM study of pre-reading dyslexic children did not report alterations in cerebellar areas (Raschle, Chang, & Gaab, 2011), while a VBM study of 11 dyslexic school children reported increased GM volume in the right anterior cerebellum after an 8-week training focused on mental imagery; articulation; and tracing of letters, groups of letters and words (Krafnick et al., 2011).

### Frontal and temporoparietal areas

We observed five areas of GM alterations in temporo-parietal areas and three in frontal areas. Generally, dyslexia (especially in relation to phonological impairments) has been linked with atypical activation of the left perisylvian fronto-temporo-parietal network (e.g. Richlan et al., 2011). However, in the meta-analysis by Richlan et al. (2012), reduced GM volumes were observed in both hemispheres: one in the left superior temporal sulcus and unexpectedly one in the right superior temporal gyrus. In the present study, all temporo-parietal and frontal GM abnormalities failed to survive corrections for multiple comparisons. Our areas in the left inferior parietal lobe extending to the supramarginal gyrus (increased GM volume for dyslexics) and in the right angular gyrus (reduced GM volume for dyslexics) are close to areas of reduced GM volume reported in the meta-analysis by Linkersdörfer et al. (2012). Six other areas were observed in parietal, temporal and frontal areas, regions close to or overlapping with areas which were reported before, either in anatomical or in functional studies.

A possibility is that unbalanced inclusion of different subtypes of dyslexia might have enhanced the finding of significant and inconsistent results in these areas in individual studies. In other words, when dyslexics exhibit different cognitive impairments, it can be expected that highly educated students apply different alternative compensation techniques leading to various clusters of augmentations or reductions. Thus, some dyslexics might try to improve their phonological abilities and others their reading abilities. This view was confirmed in a study by Peyrin et al. (2012) who observed various functional differences in both hemispheres between a young dyslexic adult with only phonological impairments as opposed to a young dyslexic adult with only an impairment of visual attention span. Another explanation for inconsistent findings in the perisylvian fronto-temporo-parietal network may be gender effects as reported by Evans et al. (2013). They observed typical left and right hemispheric alterations in men, but in women mainly right hemispheric alterations, which seems to be consistent with our observations. We only found three small areas in the left hemisphere, but three small and two large areas in the left hemisphere. As argued by Richlan et al., we should include these areas in discussions as being relevant tendencies which require further exploration.

### Limitations of this study

This study confirmed that the complex nature of dyslexia cannot easily be clarified by anatomical brain correlates. Although findings of this study contribute to the accumulating knowledge about brain correlates of dyslexia, we should also emphasise some limitations.

Although we found significant correlations, we found no significant group differences after corrections for multiple comparisons. Instead, we reported large tendencies and looked whether these tendencies correlated with behavioural measures. These tendencies were defined by clusters of 200 connected voxels with a *p* value lower than 0.05 in the VBM analysis, which is, of course, an arbitrary decision. We referred to another study which used the same threshold (Rouw & Scholte, 2010). This is a relative large threshold. A disadvantage is that small and relevant clusters may be overlooked. However, we wanted to study large tendencies without running the risk of analyzing small clusters that result from noise.

Another limitation of this study is related to the sample, which consisted of students. However, we found that using a student sample might also be an advantage. For instance, students received extensive language training at school (students with as well as students without dyslexia). This probably was related to the significant correlation between spelling abilities and reduced GM volume in the cerebellum. We argued that also other findings of the present study might be related to different compensation strategies which can assumed to be characteristic for highly intelligent students. However, as a result of this, this study could not separate brain correlates of dyslexia that result from training from brain correlates that may be present at birth.

## Conclusion

We found no significant group differences in local GM volumes between dyslexics and non-dyslexics although we used a large sample that accounted for different cognitive profiles of dyslexics. Instead, we found four significant correlations between five behavioural measures of dyslexia and local GM and total GM and WM volumes. These measures specify various specific relations with local GM volume alterations. Specifically, we found that the caudate nucleus is involved in abilities related to confusion, that the cerebellum is involved in abilities related to spelling and that both spelling and confusion are related to total WM volume. These results reveal that understanding of anatomical alterations in dyslexia is best identified when various cognitive aspects of dyslexia are acknowledged. Other findings of this study were more difficult to interpret, such as the involvement of temporo-parietal areas. Effects of sample differences cannot be ruled out, such as gender differences, age differences, differences in selection methods, differences in education and differences in experience and compensation strategies. Nevertheless, also insignificant findings might contribute across studies to accumulate evidence of brain alterations in dyslexia.
